# Utilization of dental services by preschool children: prevalence and associated factors

**DOI:** 10.1590/1807-3107bor-2024.vol38.0081

**Published:** 2024-08-26

**Authors:** Isabela da Costa GONÇALVES, Valéria Silveira COELHO, Joana RAMOS-JORGE, Priscila Seixas MOURÃO, Kaio Henrique SOARES, Maria Letícia RAMOS-JORGE, Izabella Barbosa FERNANDES

**Affiliations:** (a) Universidade Federal de Minas Gerais – UFMG, School of Dentistry, Department of Child and Adolescent Oral Health, Belo Horizonte, MG, Brazil; (b) Universidade Federal dos Vasles do Jequitinhonha e Mucuri, School of Biological and Health Sciences, Department of Dentistry, Diamantina, MG, Brazil.

**Keywords:** Child, Health Services Research, Child, Preschool, Dental Health Services, Health Services Accessibility

## Abstract

Dental associations worldwide recommend that the first dental visit should take place before 12 months of age; however, preschoolers’ utilization of dental services remains low. The aim of this study was to assess the prevalence of, and factors associated with, dental services utilization among children aged 1 to 3 years. This was a cross-sectional study carried out in the city of Diamantina, MG, Brazil, and involved a sample of 308 child-mother pairs. Mothers completed a questionnaire addressing sociodemographic and economic aspects of the family and characteristics pertaining to their child’s oral health. The clinical assessment of the children included dental caries, trauma, malocclusion, and mucosal changes. Analysis of the data comprised statistical description, application of the chi-square test, and Poisson’s regression analysis. Among the children studied, 39.6% had attended at least one dental visit in their lifetime. Children whose families had a greater number of members relying on the family’s income (PR = 1.40, 95%CI:1.04 –1.89, p = 0.028) and those with moderate/extensive dental caries (Codes 3-6 of the ICDAS; PR = 1.44, 95%CI: 1.08 –1.93, p = 0.014) exhibited a higher prevalence of dental services utilization. In conclusion, the prevalence of dental services utilization among children aged 1 to 3 years was low, and was associated with a greater number of family members relying on the family’s income, and with the occurrence of moderate/extensive dental caries.

## Introduction

Health services accessibility International dental associations recommend that children have their first dental visit before reaching 12 months of age.^1-3^ This initial visit serves to acclimatize children to the dental office environment, establish rapport with oral health professionals, and impart interactive and enjoyable guidance on preventive oral health measures.^4,5^ Despite these recommendations, the utilization of dental services by preschoolers remains notably low worldwide.^6,7^ Research indicates that despite variations in policies designed to provide access to dental care across different countries, a significant proportion ranging from 62% to 88% of preschool-aged children have never visited a dentist.^6,7^ In Brazil, despite efforts within the Public Health System to mitigate barriers to access, studies have reported a prevalence of preschool children who have never visited a dentist ranging from 56% to 87%.^8,9^

The low prevalence of dental services utilization during the early years of life can be attributed to a prevailing belief among the population that seeking dental care is only necessary when an oral problem has already manifested, particularly if accompanied by pain.^10,11^ Furthermore, there exists a misconception among parents that primary teeth do not require treatment as they will eventually be replaced by permanent teeth.^12^

Previous studies have demonstrated associations between various factors and the utilization of dental services during the early years of life. These factors include the age of the child,^8,9,13,14^ socioeconomic characteristics of the family,^9,10,11,15^ the number of family members relying on the same income,^14,16^ characteristics of parents,^7,10,13,15-18^ parents’ access to dental services,^7,9,10^ and dental care for mothers during the prenatal period.^10,17^

The first dental appointment during the early years of life plays a crucial role in maintaining optimal oral health throughout one’s lifetime. It serves as an opportunity to prevent and promptly identify various prevalent diseases that significantly impact quality of life, such as tooth decay, dental trauma, and malocclusion.^19-20^ Therefore, evaluating the prevalence and factors associated with children’s utilization of dental services during their early years is essential for identifying at-risk groups facing barriers to access.^21^ This assessment is particularly important across diverse populations, considering the unique social and cultural characteristics of each community.^22^ By understanding this information, public strategies and programs can be developed to enhance dental coverage in early childhood through multidisciplinary monitoring, thus promoting regular care for children within this age group. Therefore, the aim of the present study was to assess the prevalence of, and factors associated with, the utilization of dental services by children aged 1 to 3 years.

## Methods

### Ethical aspects

This study was approved by the Human Research Ethics Committee of the Federal University of Jequitinhonha and Mucuri Valleys (UFVJM; Approval No. 470863). Informed Consent Forms were signed by all mothers who consented to participate, both for themselves and on behalf of their children. The research adhered to the guidelines outlined in the Strengthening the Reporting of Observational Studies in Epidemiology (STROBE) statement.

### Study design and population

This cross-sectional study was conducted in the city of Diamantina, located in the northeast region of the state of Minas Gerais, southeastern Brazil, during 2017 and 2018. According to the most recent demographic census carried out in 2010, Diamantina’s Human Development Index was 0.716, and its population consisted of 45,880 inhabitants, among whom 3,013 were aged 0 to 4 years.^23^Children aged 1 to 3 years were selected from a list provided by the Municipal Health Department, comprising children who had utilized municipal public health services for vaccination in 2017. Each child on the list was assigned a numerical identifier. Subsequently, a random selection process was employed among these children until the predetermined sample size was attained. Initially, the mothers of the selected children were contacted via telephone. In instances where telephone communication was unfeasible, home visits were conducted to extend invitations for participation in the research. Should contacted mothers decline to participate in the study, an alternative child was randomly selected for inclusion.

Inclusion criteria for participation in the study required that children be between 1 and 3 years of age and free from chronic diseases or systemic conditions, as reported by their parents. Furthermore, the children’s mothers were required to accompany them to the Graduate Clinic of the Federal University of Jequitinhonha and Mucuri Valleys (UFVJM) for data collection purposes.

### Sample calculation

A sample calculation was conducted utilizing OpenEpi (Open Source Epidemiologic Statistics for Public Health) software, employing the formula for simple proportion. Considering a prevalence of 29% for access to dental care, as determined by a pilot study, and specifying a 95% confidence interval with a standard error of 5%, it was determined that a minimum sample size of 287 children was required. To compensate for potential losses, 345 children, accompanied by their mothers, were randomly selected and invited to partake in the study.

### Training and calibration

Prior to data collection, two researchers underwent training and calibration procedures. The training included diagnostic criteria for dental caries,^24^ malocclusion,^25,26^ dental trauma,^27^ and mucosal changes.^28^ This training process was coordinated by an experienced researcher, and consisted of theoretical instruction and analysis of various oral clinical scenarios using images. Calibration exercises were conducted whereby 50 children were assessed by both examiners and an experienced researcher on two separate occasions, with a one-week interval between examinations. The minimum intra-examiner and inter-examiner Kappa coefficients attained were 0.86 and 0.83, respectively.

### Pilot sudy

A pilot study involving a sample of 30 children and their mothers was conducted to assess the data collection methodology and gather information necessary for calculating the sample size for the main study. The participants from the pilot study were subsequently included in the main study, as no adjustments to the study methodology were deemed necessary.

### Data collection

Data collection took place between 2017 and 2018 at the UFVJM Graduate Clinic. Upon selection, participants and their mothers were invited to attend the clinic for data collection procedures. Initially, mothers were administered a questionnaire in the form of an interview by an interviewer blinded for the child’s clinical oral examination. Information collected in the questionnaire included the child’s age and sex, whether the child had previously visited a dentist (yes or no), a maternal report on the child’s history of toothache (yes or no), the family’s monthly income (categorized based on the Brazilian minimum wage, of approximately US$ 308.47 at the time of the study, dichotomized into > 2 or ≤ 2 minimum wages), the number of family members supported by the family’s monthly income (categorized based on the median number of family members into > 3 or ≤ 3), maternal education level (≥1 3 years, 9 to 12 years, or ≤ 9 years of schooling), maternal age (> 25 or ≤ 25 years, dichotomized according to the median age), non-nutritive sucking habits (yes or no), history of signs and symptoms associated with tooth eruption (yes or no), and potential sleep bruxism (mother’s report of the child’s grinding or clenching teeth during the night, yes or no).^29^

The second phase of data collection consisted in conducting a clinical oral examination of the children. The children were seated on a pediatric dental stretcher, with mothers assisting in holding very young children, when necessary. The examinations were performed by two examiners, and assessment was conducted under artificial light and cotton-roll isolation, following the cleaning and drying of teeth using compressed air. All procedures adhered strictly to the biosafety standards recommended by the institution.

The purpose of the clinical examination in this study was to ascertain the presence of dental caries, malocclusion, mucosal changes, and dental trauma. The International Caries Detection and Assessment System (ICDAS)^24^ was utilized to assess dental caries, and the child was considered positive for caries if at least one carious lesion classified as ICDAS codes 3, 4, 5, and/or 6 (moderate to extensive caries) was observed.^24^

The presence of malocclusion was categorized based on the identification of one of the following conditions: severe overjet (exceeding 3 mm), severe overbite (where the maxillary incisors covered 2 mm or more of the mandibular incisors), anterior open bite, and anterior or posterior crossbite.^25,26^ For children who had not yet developed occluding teeth, the classification was designated as “absence of malocclusion.”

Diagnosis of dental trauma was conducted following the criteria outlined by Andreasen et al.^27^, which involves crown discoloration, and was categorized as either absent or present.

Identification of oral mucosa changes followed the sequence delineated by Bessa et al.^28^ These conditions were recorded on a standardized clinical chart comprising 23 distinct types of conditions, encompassing both pathological processes and variations of normality, and were categorized as either present or absent.

### Statistical analysis

The data was analyzed utilizing the Statistical Package for the Social Sciences program (SPSS for Windows, version 22.0, SPSS Inc., Chicago, IL, USA). A statistical description of the sample was conducted, and the chi-square test was employed for inferential analysis. Additionally, Poisson’s regression analysis with robust variance was conducted to examine associations between the utilization of dental services and factors pertaining to sociodemographic and economic characteristics of the family, as well as characteristics related to the child’s oral health and oral clinical conditions. Variables deemed crucial to the theoretical model, which could potentially act as confounding factors affecting the results, were retained in the analysis to adjust the final model. Prevalence ratios (PR) along with their corresponding 95% confidence intervals (CI 95%) were calculated.

## Results

Out of the 345 pairs of children/mothers invited to partake in the study, 37 pairs failed to attend the data collection sessions. Consequently, 308 child/mother pairs, comprising 89.3% of the initially invited participants, participated in this cross-sectional study. Among the 308 children assessed, 39.6% had visited a dentist at least once in their lifetimes. Most of the children were female (54.9%), with 2 years being the most prevalent age group (37.3%). Concerning maternal education, the largest proportion fell within the range of 9 to 12 years of schooling (51%). A total of 190 families (61.7%) reported a monthly family income of up to two minimum wages.

Table presents the statistical description of the data on the prevalence of dental services utilization by children according to sociodemographic and economic variables, maternal characteristics, and other aspects related to the child. After adjustment, the final Poisson regression model (also depicted in Table 1) revealed that a greater number of members relying on family income remained significantly associated with dental services utilization (PR = 1.40, 95%CI: 1.04–1.89), along with the finding of moderate to extensive dental caries (PR = 1.44; 95%CI: 1.08–1.93).

**Table:** Statistical description of the data found on the prevalence of dental service utilization by children, according to sociodemographic and economic variables, maternal characteristics, and pertinent aspects relating to the child. Both unadjusted and adjusted Poisson regression analyses were performed to evaluate the association between dental service utilization and independent variables (n = 308).

Covariates	Prevalence of dental services utilization			Not adjusted PR	p-value	Adjusted PR	p-value
	
	yes	no	total	(95%CI)		(95%CI)	
Sex							
Feminine	68 (40.2)	101 (59.8)	169 (54.9)	1			
Masculine	54 (38.8)	85 (61.2)	139 (45.1)	0.97 (0.73-1.27)	0.805	-	NS
Age (years)							
1	34 (33.3)	68 (66.7)	102 (33.1)	1			
2	46 (40.0)	69 (60.0)	115 (37.3)	1.20 (0.84-1.71)	0.313		
3	42 (46.2)	49 (53.8)	91 (29.5)	1.38 (0.97-1.97)	0.071	-	NS
Maternal education (years of schooling)							
≥ 13	43 (43.4)	56 (56.6)	99 (32.1)	1			
9–12	58 (36.9)	99 (63.1)	157 (51.0)	0.85 (0.63-1.15)	0.296		
≤ 9	21 (40.4)	31 (59.6)	52 (16.9)	0.93 (0.62-1.39)	0.721	-	NS
Family’s monthly income							
> 2 minimum wages	44 (37.3)	74 (62.7)	118 (38.3)	1			
≤ 2 minimum	78 (41.1)	112 (58.9)	190 (61.7)	1.10 (0.82-1.47)	0.515	-	NS
Number of family members relying on family income							
≤ 3	42 (32.3)	88 (67.7)	130 (42.2)	1		1	
> 3	80 (44.9)	98 (55.1)	178 (57.8)	1.39 (1.03-1.87)	0.030	1.40 (1.04-1.89)	0.028
Maternal age							
≤ 25	57 (36.3)	100 (63.7)	157 (51)	1			
> 25	65 (43.0)	86 (57.0)	151 (49)	0.84 (0,64-1.11)	0.228	-	NS
Non-nutritive sucking habit							
No	88 (42.3)	120 (57.7)	208 (67.5)	1			
Yes	34 (34.0)	66 (66.0)	100 (32.5)	0.80 (0.59-1.10)	0.175	-	NS
Toothache							
No	89 (38.4)	143 (61.6)	232 (75.3)	1			
Yes	33 (43.4)	43 (56.6)	76 (24.7)	1.42 (1.07-1.89)	0.015	-	NS
Teething disturbances							
No	9 (42.9)	12 (57.1)	21 (6.8)	1			
Yes	113 (39.4)	174 (60.6)	287 (93.2)	0.92 (0.55-1.54)	0.747	-	NS
Potential sleep bruxism							
No	96 (39.5)	147 (60.5)	243 (78.9)	1			
Yes	26 (40.0)	39 (60.0)	65 (21.2)	1.01 (0.72-1.42)	0.942	-	NS
History of dental trauma							
No	91 (39.6)	139 (60.4)	230 (74.7)	1			
Yes	31 (39.7)	47 (60.3)	78 (25.3)	1.00 (0.73-1.38)	0,978	-	NS
Presence of malocclusion							
No	88 (39.3)	136 (60.7)	224 (72.7)	1			
Yes	34 (40.5)	50 (59.5)	84 (27.3)	1.03 (0.76-1.40)	0.848	-	NS
Changes in the mucosa							
No	103 (39.0)	161 (61.0)	264 (85.7)	1			
Yes	19 (43.2)	25 (56.8)	44 (14.3)	1.11 (0.76-1.60)	0.592	-	NS
Dental caries							
Absent	56 (32.0)	119 (68.0)	175 (56.8)	1		1	
Present	66 (49.6)	67 (50.4)	133 (43.2)	1.55 (1.18-2.04)	0.002	1.44 (1.08-1.93)	0.014

PR: prevalence ratio; CI: confidence interval; NS: non-significant associations (p > 0,05).

## Discussion

The main findings of this study was that a minority of children had attended dental visits before reaching the age of 3 years. The key factors associated with the utilization of dental services during the early years of life were greater number of family members relying on family income and the finding of moderate to extensive dental caries.

The limited utilization of dental services among preschool children is a trend observed in various population-based studies conducted worldwide. These studies, spanning diverse regions and countries, have consistently reported comparable prevalence rates of children who have never visited a dentist, aligning closely with the findings of the present study.^6-10,13,17,30^ This collective evidence underscores the widespread prevalence of preschool children who lack dental care access, despite variations in access strategies to dental services across different countries/regions and the diverse sociodemographic and cultural attributes of the populations under evaluation.

The low prevalence of dental services utilization among preschool children may be attributed to several factors. Firstly, it could stem from parents’ limited awareness regarding the optimal age for a child’s first dental visit.^18^ Additionally, there may exist a prevailing belief within the population that primary teeth, being temporary, do not require dental care, as they will eventually be replaced by permanent teeth.^12^ Furthermore, the absence of incentive and informational programs concerning early childhood dental care could diminish the demand for these services.^18^ Effective implementation of such programs necessitates the development of strategies aimed at motivating the population to seek dental services for preventive purposes, as well as educating them about the significance of oral health care during infancy and the potential consequences of neglecting oral issues pertaining to primary teeth.

Our study revealed that children afflicted with dental caries exhibited a higher prevalence of dental services utilization, a finding consistent with previous research.^8,9,14^ Despite contemporary efforts to promote a more holistic and preventive approach to healthcare, our findings suggest that the demand for dental care remains largely tethered to the biomedical model, wherein the focus primarily revolves around disease management.^31^ Consequently, seeking dental care is often prompted by the presence of disease rather than prioritizing health maintenance.

In this study, only “obvious” caries lesions or cavitated lesions were assessed. These types of lesions are more easily discernible by parents^32^ and have a considerable impact on a child’s quality of life.^33^ Including “non-obvious” caries lesions or non-cavitated lesions in the assessment of caries prevalence may lead to an overestimation of the extent of dental caries experience. Particularly in primary teeth, the prevalence of caries can rise to as high as 95.6% when “non-obvious” caries lesions are taken into account.^34^

Another noteworthy finding of the present study is that children from families with a greater number of members relying on the family’s income exhibited a higher prevalence of dental services utilization. Previous research has indicated that children from larger families tend to have a heightened occurrence of dental caries, alongside a detrimental impact on Oral Health-Related Quality of Life (OHRQoL).^33,34^ Within large families, the caregivers’ attention may be divided among other family members, potentially relegating oral hygiene and dietary care for the child to a secondary priority. This dynamic can exacerbate oral health conditions, consequently increasing the demand for dental care, particularly for curative interventions.

However, it is worth noting that some studies have reported contrasting findings regarding the prevalence of dental services utilization among children from larger families.^14,15^ For instance, one study found that the utilization of dental services was negatively associated with having more than four children in the family, in comparison with families having only one child.^16^

Among the socioeconomic factors investigated, our study did not reveal any association between maternal education, family income, and the utilization of dental services by children aged 1 to 3 years. However, previous research has demonstrated a consistent link between socioeconomic status and the frequency of dental visits.^9-11,14,15,17,35,36^ Several studies have indicated that children from families of low socioeconomic status tend to visit the dentist less frequently,^9-11,14,15,17^ primarily due to several risk factors that impede access to healthcare resources and services, including psychosocial and environmental factors, as well as material deprivation.^15^

Conversely, other studies have reported an inverse association, highlighting that children from families with low socioeconomic status, owing to their elevated prevalence of oral diseases, often visit the dentist more frequently for curative interventions.^35,36^ The absence of such an association in our study may be attributed to contextual factors specific to the city of Diamantina, where the research was conducted. In this city, socioeconomic disparities concerning oral health are ameliorated by the provision of free and high-quality dental services offered by the Unified Health System (UHS), accessible at Basic Health Units (BHUs) and university-affiliated facilities. These institutions cater to a significant portion of the population with dental needs, thereby mitigating disparities in access to dental care based on socioeconomic status.

Previous studies have explored the relationship between a child’s age and the utilization of dental services, and have found a higher prevalence of dental services utilization among older preschoolers.^8,13,14^ However, no association between these variables was observed in our study. This lack of association may be attributed to the fact that our study exclusively focused on younger children, up to 3 years old, whereas previous studies included preschoolers up to 5 years old.

It is plausible that oral problems exhibit a cumulative effect, becoming more conspicuous as children age.^13^ Consequently, parents may be more inclined to seek professional assistance as their children grow older, particularly with the emergence of more discernible signs and symptoms of oral diseases. This phenomenon likely elucidates the association between age and the utilization of dental services observed in previous studies.

In our study, the association between toothache and the utilization of dental services was not retained in the multivariate analysis. This contrasts with findings from previous studies, which indicated higher dental services utilization among individuals experiencing toothache.^10,11,37^

One plausible explanation for this discrepancy may relate to the age of the children evaluated in our study. Younger children may have more difficulty articulating their discomfort, making the interpretation of symptoms by family members more subjective.^38^ Consequently, the association between toothache and dental services utilization may not be as pronounced in this age group.

Moreover, it is important to underscore that seeking dental care solely in response to toothache is not ideal. Doing so may inadvertently associate dental visits with pain and discomfort,^7^ potentially fostering dental anxiety in children. Instead, preventive dental visits should be prioritized to prevent oral conditions from progressing to more advanced stages, which may necessitate more invasive and distressing interventions.^30^

Other oral changes evaluated in our study, including malocclusion, non-nutritive sucking habits, and bruxism, were not found to be associated with the utilization of dental services by preschoolers. This finding is consistent with previous research, which similarly reported no association between bruxism,^30^ malocclusion,^11,30^ dental trauma,^17^ and the utilization of dental services.

Parents may have limited proficiency in detecting dental conditions in their children, particularly when the condition does not elicit pain or discomfort.^33,40^ Consequently, parents may only seek dental care for their children in cases of more severe oral alterations that are aesthetically or functionally significant. This selective approach to seeking dental care may be influenced by the perception that only serious oral conditions warrant professional attention.

While this study provides valuable and original evidence, its cross-sectional design precludes the establishment of causal relationships between the variables investigated. Future research endeavors should aim to elucidate the factors underlying parental decisions to forego dental care for their children. Additionally, there is a need to assess parents’ awareness regarding the significance of early dental visits for children. Investigating these aspects will contribute to a more comprehensive understanding of the barriers to dental care utilization among young children and facilitate the development of targeted interventions aimed at improving access to dental services during the critical early years of life.

This information can serve as a foundational framework for the development and implementation of public health policies geared towards promoting early dental care seeking behaviors. Such policies have the potential to yield direct benefits to the targeted population by enhancing access to dental services and fostering parental awareness regarding the preventive aspects of dental care, rather than solely focusing on therapeutic interventions.^9^

It is crucial to underscore the role of all healthcare professionals as advocates for oral health promotion. Given that children often have initial contact with pediatricians or nurses before visiting a dentist,^16^ it is imperative to emphasize multidisciplinary collaboration to facilitate referrals and encourage dental service utilization. The integration of oral health initiatives within maternal and child health programs holds promise for increasing the regular utilization of dental services, thereby contributing to the enhancement of oral health outcomes during early childhood.^39^

## Conclusion

The present study revealed a low prevalence of dental service utilization among children aged 1 to 3 years. Significant associations were observed between dental services utilization and two key factors: the presence of moderate to extensive dental caries and a higher number of family members relying on the family’s income.
